# Editorial: Genetic Basis of Thermoregulation in Livestock

**DOI:** 10.3389/fvets.2022.839612

**Published:** 2022-03-11

**Authors:** Rajib Deb, Vinicius De França Carvalho Fonsêca, Rita Payan-Carreira, Veerasamy Sejian, Angela M. Lees

**Affiliations:** ^1^Animal Health Division, ICAR-National Research Center on Pig, Guwahati, India; ^2^Division of Physiology, Federal University of Paraíba, João Pessoa, Brazil; ^3^Comprehensive Health Research Centre and Department of Veterinary Medicine, University of Evora, Évora, Portugal; ^4^Centre for Climate Resilient Animal Adaptation Studies, ICAR-National Institute of Animal Nutrition and Physiology, Bengaluru, India; ^5^School of Environmental and Rural Science, University of New England, Armidale, NSW, Australia

**Keywords:** thermoregulation, genetics, cattle, pig, SNPs

Livestock production will continue to play an important role in securing the livelihoods of many small-scale farmers and pastoralists in the coming years. We must concede the superior traits of livestock breeds emphatically and scientifically under changing climatic parameters so that future generations can benefit from the priceless genetic resources in terms of global food security and livelihoods in meeting the demand for energy, protein, and other critical nutrients.

One of the major environmental factors that influence the health and productivity of livestock populations is temperature or heat stress. The increase in global temperatures puts heat stress at the center of ever-growing concern regarding livestock populations across tropical and temperate zones. Various livestock species and breeds present differential thermoregulatory capacities to challenge thermal stress based on anatomical and physiological differences. However, genetic components, variations in gene expression, and molecular mechanisms underlying acclimation and adaptation to heat stress in livestock have yet to be extensively documented. The goal of this Research Topic is to highlight recent advances in molecular mechanisms governing climate resilient livestock production by highlighting research, datasets, and methodology-based manuscripts, as well as high-quality strategic reviews. This Research Topic attracted submission of articles highlighted the genetic basis of thermoregulatory mechanisms in livestock especially dairy animals.

The current trend in global warming puts livestock productivity and welfare in jeopardy. Identifying climate-resilient animals is therefore critical for sustainable production, despite the negative effects of thermal stress.

Estimation of the genetic parameters for thermoregulation traits defines the ability to adapt to extreme thermal environment stimuli. The identification of heat tolerant genotypes is challenging because the responses to heat stress are complex and variable, but also because it would request a definition of what is a heat-resilient animal and whose traits should we look for, or how much close are the genetic indicators of the productive performance of farm animals. This difficulty could explain why there is no practical commercial breeding program that includes thermoregulation traits in the selection index. Therefore, there is still the need to produce growing evidence not only of genetic traits that would suggest a thermal tolerance core profile in an individual or breed, but also how can they be used to hint at differences in adaptative strategies that represent environmental copying with heat stress.

To identify climate-resilient dairy cows, researchers used a variety of genomic tools. Next-generation sequencing (NGS), microarray technology, whole transcriptome analysis, and genome-wide association studies (GWAS) are examples of recent advances in molecular biology that can be used to quantify the molecular mechanisms that govern dairy cows' climate resilience capacity. Silpa et al. critically reviewed the application of various genomic tools as well as statistical models for development of climate resilient dairy cattle herd. They projected data on the effects of heat stress on dairy cow milk production as well as the various genomic tools available to identify climate-resilient dairy cows. Through a marker-assisted selection programme, such candidate genes can be incorporated into dairy cattle's climate resilience capacity. Researchers are now focusing on incorporating thermo-tolerance and low methane emission traits into breeding programmes that were previously focused on productive traits in light of the current climate change scenario. A holistic breeding approach like this would ensure long-term livestock production.

Selection signatures can be used to detect potential loci and candidate genes that have undergone positive selection in complex production traits in livestock. To develop climate-resilient animals, these identified biomarkers can be incorporated into existing breeding policies using genomic selection. Based on dense SNP data, Freitas et al. investigated genetic diversity and unravel genomic regions potentially under selection for heat and/or cold tolerance in 32 global cattle breeds, with a focus on Chinese local cattle breeds adapted to divergent climatic conditions, Datong yak (*Bos grunniens*; YAK), and Bali (*Bos javanicus*). Heat-shock proteins, oxygen transport, anatomical traits, mitochondrial DNA maintenance, metabolic activity, feed intake, carcass conformation, fertility, and reproduction were all identified as important genomic regions and candidate genes by selection signatures. Thermal tolerance has a complex polygenic inheritance nature, as expected given the various mechanisms involved in thermal stress response, based on the large number of genomic regions identified.

Fang et al. investigated the possibility of simultaneously developing high-yielding and thermotolerant cattle using marker-assisted selection. After cold and heat stress treatments, they found 29 and 41 significantly differentially expressed genes (DEGs) (fold change 1.2-fold and *P* <0.05), respectively. The *HIF1A* gene's mRNA expression was upregulated during cold stress, while the *EIF2A, HSPA1A, HSP90AA1*, and *HSF1* genes were downregulated after heat exposure, according to their validation studies at the cellular and individual levels.

The detection of genetic variants in heat shock protein genes and the resultant phenotypic traits can potentially be used to determine thermo tolerance or resistance of livestock species to thermal stress. Onasanya et al. investigated the effect of a single nucleotide polymorphism (SNP) in the heat shock protein 70 (Hsp70) gene on heat stress tolerance in various Nigerian zebu cattle breeds. They discovered multiple SNPs in Hsp70 using a quantitative real-time/high-resolution melting (HRM) assay. Heterozygous animals had a lower heat tolerance coefficient (HTC), indicating that they were less able to withstand heat stress than homozygous animals.

The genetic basis of thermoregulation in pigs was critically reviewed by Gourdine et al. They present a summary of what is currently known about the genetics of thermoregulation in pigs. They also talked about the various phenotypes that can be used in genetic studies, as well as the differences in thermoregulation between pig breeds and the inheritance of thermoregulation traits. This review also considers the ongoing challenges that must be overcome in order to improve pig heat tolerance. Implementing thermoregulatory traits directly in a conventional pig breeding programme is difficult, first because it is difficult to define thermal tolerance directly in terms of measurable traits, second because it is difficult to measure these appropriate phenotypes routinely and technically easily under heat stress conditions, and third, as far as we know, because there is no quantification of economic weights of thermoregulatory traits. However, genomic innovations and precision selection will undoubtedly be used in future genetic studies on pig thermoregulation. They also revealed existing individual variations among groups suggesting that some markers might be available to be used in the genetic selection of thermotolerant animals.

As a result, genetic selection opens up a new avenue for selecting more climatically resilient livestock species. The studies in this Research Topic provide excellent examples of the genetic basis of livestock thermoregulation and their utility in the development of biomarkers.

## Author Contributions

All authors contributed to manuscript revision, read, and approved the submitted version.

## Conflict of Interest

The authors declare that the research was conducted in the absence of any commercial or financial relationships that could be construed as a potential conflict of interest.

## Publisher's Note

All claims expressed in this article are solely those of the authors and do not necessarily represent those of their affiliated organizations, or those of the publisher, the editors and the reviewers. Any product that may be evaluated in this article, or claim that may be made by its manufacturer, is not guaranteed or endorsed by the publisher.

